# Resilience in Teachers: Validation of the Spanish Version of the CD-RISC10© Scale in Early Childhood, Primary and Special Education Teachers

**DOI:** 10.3390/ijerph191711020

**Published:** 2022-09-03

**Authors:** Raquel Flores-Buils, Antonio Caballer-Miedes, Rosa Mateu-Pérez

**Affiliations:** 1 Department of Developmental, Educational and Social Psychology and Methodology, Universitat Jaume I, 12071 Castellón, Spain; 2 Department of Pedagogy and Didactics of Social Sciences, Language and Literature, Universitat Jaume I, 12071 Castellón, Spain

**Keywords:** resilience, CD-RISC 10©, teachers, psychometric properties, validity, reliability

## Abstract

Schools are a fundamental context for processes of promotion and generation of resilience, since they focus not only on aspects of academic development, but also of personal and social development. Resilience in the teacher has a dual function. On the one hand, as resilient professionals, teachers can foster the development of resilience in their students; on the other hand, teaching resilience facilitates their own personal and professional well-being. Despite research highlighting the benefits of resilience in education, there is scarce research focused on assessing resilience in teachers. Thus, the aim of this paper is to analyze the psychometric properties of the Spanish version of Connor and Davidson’s 10-item resilience scale (CD-RISC 10©) in a sample of 290 teachers. A confirmatory factorial analysis (CFA) is performed, which shows that the 10 items on the CD-RISC 10© Resilience Scale form a one-dimensional structure, with high reliability, McDonald’s Omega coefficient (ω = 0.80) and Cronbach’s alpha coefficient (α = 0.87). The results obtained in this work support the use of the scale for the assessment of resilience in teachers of Infant, Primary and Special Education, which is considered very useful, not only to know their degree of resilience, but also to assess the effectiveness of training programs.

## 1. Introduction

Resilience is a dynamic and multidimensional construct that refers to the ability of personal systems to successfully cope with or recover from adverse situations; it is associated with positive growth and overcoming challenges [1], so it implies a positive adaptation of the person, despite past and/or daily difficult situations [2,3].

Resilience has three essential components: the notion of adversity, trauma, risk or threat to human development; positive adaptation or overcoming adversity; and the process that considers the dynamics between emotional, cognitive and sociocultural mechanisms that influence human development [4]. Therefore, it should be taken into account that it is not a fixed trait or attribute, as one is not born resilient nor is it acquired during development, but rather it is an interactive process between people and their environment when facing a risk situation [5,6], which is aimed at positive growth and significant well-being in each situation and stage of life [7,8].

New perspectives highlight the fact that resilience can be developed as a dynamic process [6,9,10], where protective and risk factors intervene. Protective factors can be individual (qualities, such as independence, introspection, ability to relate, initiative, humor, creativity and morality) [10] and environmental factors, which refer to those intrafamilial and extrafamilial characteristics that optimally condition the development of individuals. Risk factors refer to those characteristics of a person (such as family history of alcoholism, lack of impulse control or disabilities), interpersonal aspects (such as the chronic illness of a family member or a situation of family conflict) or environmental aspects (such as a situation of poverty) that are linked to a high probability of damaging their health [11,12]. In the field of teacher resilience, there are studies that establish, as protective factors, self-efficacy, commitment, motivation, sense of achievement, professional identity [13,14,15], as well as the good behavior of students [16] and the support of colleagues and the institution [17]. As risk factors in teachers, studies point to professional burnout, lack of resources [18], low student motivation [16], lack of support and demands perceived by educational policies [14].

Several studies highlight the suitability of educational centers for building resilience, as they are contexts that focus on the integral development of individuals and communication spaces where positive bonds can be established [14,19,20].

In educational centers, students are affected by conditions of poverty, parental unemployment, illness, personal loss, homelessness, abuse, bullying among peers, among other adverse situations. In addition, current global policies establish an Inclusive Education, following the provisions of the Global Education Agenda 2015–2030 [21], responding to the fourth Sustainable Development Goal (Quality Education), specifically, to the action of ensuring inclusive, equitable and quality education. This implies that students with functional diversity or special educational needs should attend regular classrooms. In this regard, it should be taken into account that a functional diversity, a disorder or a specific difficulty is an adverse circumstance, over which the student has little control [22]. The student may experience academic difficulties, frustration, feelings of being different and peer rejection, resulting in low self-esteem and lack of confidence. In this situation, the high level of stress associated with the school environment means that the school itself may be a risk factor for a child with special difficulties [23]. In fact, studies, such as that of Bender, Rosenkrans and Crane [23], indicate that these students experience more stress in the school environment, which often manifests itself in internal disorders, such as anxiety and depression.

In this context, teachers sometimes do not feel that they have sufficient skills to deal with this student body that is faced with any type of adversity or problematic situation, which can lead to feelings of ineffectiveness and episodes of discomfort [24,25]. Teachers must face multiple challenges on a daily basis and constantly adapt to perform their role in a changing context [26], which can lead to fragile mental health, professional burnout or stress [27]. In addition, some organizational factors, such as workload, lack of institutional support, negative classroom climate, weak autonomy and control at work and differences arising with peers, act as a barrier and hinder school improvement [27,28,29].

Teacher resilience is an individual, relational and collective process [30], which includes psychological characteristics, such as kindness, emotional stability and control, optimism and frustration tolerance [19], among other attributes, such as altruism, sense of humor, patience and enthusiasm [31], as well as contextual support, with job recognition and maintaining good peer relationships [32].

Thus, resilience in teachers has a double aspect [33]. On the one hand, as resilient professionals, the promotion of resilience in students, influencing through the teacher’s educational practices, both in prevention (working with students to strengthen individual qualities of resilience, providing support and affection and promoting networking by involving families) as well as in intervention, when the teacher works with students who are facing an adverse situation of any kind, offering them guidelines and coping strategies and showing the importance of the environment, becoming a tutor or guide for them [34,35,36]. This strengthening of resilience reduces the likelihood of the emergence of problems associated with physical and mental health, as well as school dropout [37]. However, this fostering of resilience in students can only occur if the teacher has developed resilience mechanisms, since this has an important influence on the strengthening of students’ skills [20].

On the other hand, teacher resilience favors the achievement of school goals [38] and personal and occupational well-being [39], as it strengthens intrinsic motivation despite difficulties [24], a strong sense of purpose [40], self-efficacy [41], coping strategies [13,28] and dispositional optimism [42,43]. Resilience modulates teacher distress, allowing them to overcome or adapt to stressful situations, leading to greater dedication, motivation and vigor in the goal of achieving learning and responding to the demands required by the teaching profession [18].

Despite the benefits of resilience, research on teacher resilience is at an early stage [44,45]. Some recent studies that have focused on analyzing teacher resilience reveal moderate to high levels [25,46,47]. Regarding the sociodemographic variables of teachers that have been analyzed to examine their relationship with resilience, some studies did not observe significant differences as a function of gender, age, years of experience and educational level at which teaching is provided [13,25,48]. On the contrary, there are studies that found a higher level of resilience in women than in men [47], higher level of resilience in teachers with a greater number of years of teaching experience versus novice teachers [46,49] and higher level of resilience in teachers who had higher or additional studies (master’s vs. bachelor’s degrees or with doctoral studies) [25,50]. Thus, the need for further exploration into variables that may influence the development of teacher resilience arises. Considering the benefits of teacher resilience in the educational context, its evaluation is considered essential in order to promote these qualities.

In the literature, there are only a few scales of teacher resilience and these are designed and evaluated for specific samples, such as the Escala de Resiliencia Docente [51], that took into account the specific characteristics of the Peruvian situation and the Multidimensional Teacher Resilience Scale [52], specific for Portuguese secondary school teachers. Among the validated adult resilience measurement instruments (The Resiliency Scale [53]; Dispositional Resilience Scale [54]; SV-RES Scale [55]) is the 10-item Connor–Davidson resilience questionnaire, which has shown good psychometric properties and usefulness for the measurement of resilience in different population groups [56,57,58,59,60,61] and, in particular, in Spanish samples [62,63,64]. In all the studies carried out, a unifactorial model was observed [56,57,58,59,60,61,62,63,64]; however, Aloba et al. [65] observed that resilience was better explained by a bifactorial model, which, according to the authors themselves, may be because, as the concept of resilience is not a single construct, differences may be observed in very specific samples, such as theirs.

Thus, CD-RISC-10 is one of the most widely used scales and we consider it to be a very useful assessment tool because it is reliable and valid and because it allows resilience to be assessed quickly and easily.

The novelty of the present work is that, to the best of our knowledge, there is no evidence of the administration and validation of the CD-RISC 10© in a sample of teachers.

Therefore, the aim of the present study is to evaluate the psychometric characteristics of the Spanish version of the CD-RISC 10© questionnaire in a sample of Early Childhood, Primary and Special Education teachers and to analyze whether differences are found in the level of resilience of teachers according to sex, type of school, years of teaching and the educational level where they teach.

## 2. Materials and Methods

### 2.1. Participants

The sample was made up of 290 teachers from 13 schools in Spain. Twelve of them were ordinary schools and one was a special education school. Further, 78% of the teachers were women and 22% men. The age range was between 25 and 59 years (M = 44; SD = 15.8). The participants were teachers from different educational stages and their years of teaching experience were taken into account. Table 1 shows the distribution of the sample according to some of the socio-demographic variables of the participants.

### 2.2. Instruments

#### 2.2.1. Contextual Data

The researchers asked teachers for interesting socio-demographic data to better understand the characteristics of the participants. This information includes: the sex of the teacher, type of school they work in, educational stage in which they teach and the number of years of teaching experience.

#### 2.2.2. Connor–Davidson Resilience Scale (CD-RISC 10©)

Resilience is assessed with a scale formed by items 1, 4, 6, 7, 8, 11, 14, 16, 17 and 19 [56] based on the Resilience Scale of Connor and Davidson© [66]. The response form for the items is a Likert-type scale (0 = never to 4 = always), where each participant has to indicate to what extent each of the statements was true for him/her during the last month. Total scores range from 0 to 40, where a higher score expresses greater resilience.

#### 2.2.3. Teacher as Resilient Professional (C-DPR)

C-DPR [67] assesses teachers’ competencies as resilient professionals. It is composed of 10 items and its factorial structure is composed of three factors that measure beliefs (factor 1), knowledge (factor 2) and resources (factor 3) on resilience. The reliability of the scale is high (α = 0.80) and it has good psychometric properties. The response form of the items is a Likert-type scale (from 0 to 4). Total scores range from 0 to 40, where a higher score indicates greater competence as a resilient professional.

### 2.3. Procedure

The research team contacted Professor Jonathan Davidson to request his permission to use the CD-RISC 10© scale. After sending the agreement contract, the study profile and the copyright payment, he sent us the Spanish version of the scale authorized by them, as well as the user’s manual.

A convenience sampling strategy was used and 13 schools agreed to participate in the study. The researchers met with the management of each of the schools to explain the objectives of the study and the procedure to be followed. After the approval of the management team of each of the schools, the researchers met in a classroom of the school, provided by the school itself, with the teachers to explain the objective of the research and the content of the scale. In addition, they were given an informed consent document to fill out. They were informed that participation in the study is voluntary.

The research took into account the ethical standards of the 1964 Declaration of Helsinki and its subsequent modifications. The study is authorized by the Ethics Committee of the university to which the researchers belong (code: CD/17/2022) and permissions were requested from the regional government.

### 2.4. Data Analysis

The descriptive analysis and the evaluation of reliability through internal consistency was carried out with IBM SPSS^®^Statistics version 27 (IBM Corporation, New York, NY, USA). The evaluation of the internal structure of the CD-RISC 10© was carried out through a confirmatory factor analysis (CFA) with the EQS 6.3 program [68]. Following Kenny [69], the sample of 290 participants was considered adequate for the analysis due to the correlations between the items. Because the items of the scale have a Likert format, the analyses are performed through the matrix of polychoric correlations and estimation by maximum likelihood and robust estimators. Goodness of fit is assessed through the following indices: the robust Satorra–Bentler statistic, the comparative fit index (CFI), the non-normed fit index (NNFI) and the root mean square error of approximation (RMSEA) [70,71,72]. The reliability of the questionnaire is examined through the McDonald omega coefficient [73] and Cronbach’s alpha [74], with the factor loadings obtained in the CFA and the corrected item correlations are also calculated. To analyze the correlation between CD-RISC and other indicators of resilient aspects in teachers (convergent validity), Pearson’s correlation is used. It is found that the data follow a normal distribution (Z = 0.765; *p* = 0.602). Subsequently, to determine the resilience profile of the teachers as well as the differences between sociodemographic variables in the sample, descriptive analyses (mean, standard deviation) and statistical analyses are performed using Student’s *t*-test and analysis of variance (ANOVA) with corresponding size effects (Cohen’s d and Eta squared).

## 3. Results

### 3.1. Confirmatory Factor Analysis

To determine the factor structure of the scale that best fits our sample, two models were examined. The first model (M1) analyzed a unifactorial structure, with all items. The second model (M2) exactly replicated the bifactorial structure obtained for the scale in a previous study: F1 (hardiness) items 4, 6–10 and F2 (motivation) items 1, 2, 3 and 5 [66].

To examine the fit of the data to the factorial solution of each of the models, the following indices are obtained: Satorra–Bentler Chi-square, the comparative fit index (CFI), the non-normalized fit index (NNFI) and the root mean squared error of approximation (RMSEA) (see Table 2).

**Figure 1:**
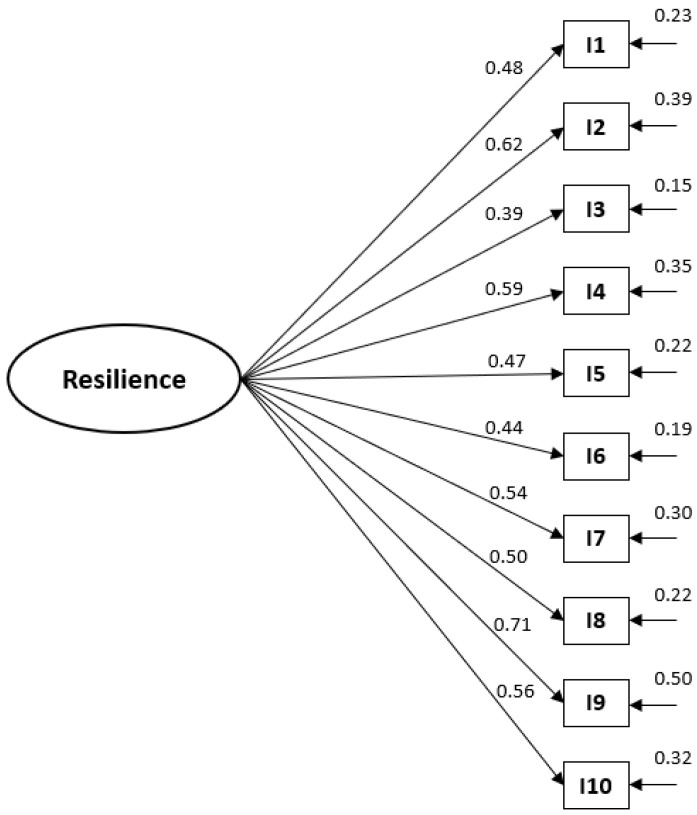
CD-RISC 10© model with standardized parameter values.

As can be seen, the data from the bifactorial model do not fit adequately, being the unifactorial model the one that fits with the following results: robust Satorra–Bentler statistic = 45.67; *p* = 0.07, the CFI = 0.97, the NNFI = 0.98 and the RMSEA = 0.036. The results indicate an acceptable fit of the data to the unidimensional model (see Table 2). The model can be seen in Figure 1, which includes the standardized parameter estimates, all of which are statistically significant.

### 3.2. Reliability

The descriptive analysis of the items, the corrected correlations and the correlations between the items can be seen in Table 3 and Table 4. Subsequently, the reliability of the scale is analyzed through Cronbach’s alpha coefficient (α = 0.87). We estimated the changes in Cronbach’s alpha if any item was eliminated. The results (see Table 3) show that reliability decreases if any item is deleted. Therefore, we cannot distinguish any weak items in the scale. In addition, we calculated McDonald’s omega to estimate the overall factor saturation in the test (ω = 0.80).

### 3.3. Convergent Validity

The correlations between CD-RISC-10 and the questionnaire on teachers’ competencies as resilient professionals are shown in Table 5.

Significant positive correlations were found between the CD-RISC-10 scores with each of the factors (beliefs, knowledge and resources) as well as with the total C-DPR score.

### 3.4. Descriptive Statistics

The data extracted after the application of the scale indicate that the mean resilience of our teachers is M = 28.71 (SD = 4.11).

Table 6 shows the means and standard deviations in resilience of the teachers, taking into account the different sociodemographic variables measured.

Regarding the sex variable, the data extracted from the *t*-test show that there is no significant difference in the level of resilience between men and women (t (277) = 0.953; *p* = *0*.341; *d* = *0*.542). As can be seen in Table 5, despite there being no significant difference, the mean score of men is higher than that of women.

ANOVA test is performed for the variables type of school, educational stage and years of experience.

If we focus on the type of school where teachers work, the data show that there are no significant differences between the resilience of teachers working in public, concerted or private schools (*F* (2.287) = 1.452; *p* = *0*.236; η^2^ = 0.060). It is found that the highest resilience mean is that of teachers working in private schools, followed by concerted and publicschools.

No significant difference was found in the mean resilience of teachers working in different educational stages (*F* (2.259) = 0.260; *p* = *0*.771; η^2^ = 0.082). It is found that special education teachers have the lowest mean, with primary education teachers having the highest mean in resilience.

Finally, if we focus on the variable years of experience, no significant differences are observed either (*F* (2.287) = 1.452; *p* = *0*.236; η^2^ = 0.085), with higher means being found the greater the teaching experience.

## 4. Discussion

The aim of the present study is to evaluate the psychometric properties of the CD-RISC 10© scale in a sample of pre-school and primary school teachers. The results confirm the unifactorial structure of the scale, similar to other previous results [56,57,58,59,60,61,62,63,64]. The data also show adequate reliability and homogeneity indices, so it is considered to be a suitable assessment instrument to measure the resilience profile of Early Childhood, Primary and Special Education teachers.

The correlations between the level of teacher resilience measured with the CD-RISC-10 with the beliefs, knowledge and resources that teachers possess in resilience (C-DPR) confirm the relationship between the resilience measure and the competencies of teachers as resilient professionals. This is in line with studies that highlight that resilience is not a fixed trait or attribute, but a state variable that can be developed so it is considered important that teachers receive training in resilience [19,20,47].

No significant differences were found in the level of resilience of teachers, taking into account the different sociodemographic variables measured (with moderate effect sizes), a fact that coincides with previous studies that also found no differences in the same variables [13,25,48].

Despite not finding significant differences, with respect to the sex variable, a higher mean resilience was obtained in men. In the literature, there are some studies that obtained a higher level of resilience in men [59] and other studies in women [47], so we can intuit that sex does not have a direct relationship with the development of resilience, although further research is needed.

Regarding the resilience of teachers according to the type of center, the highest averages are those of teachers in private and subsidized centers, possibly because they have greater academic and personal resources than public centers, which could act as a protective factor.

If we look at the data obtained regarding the resilience of teachers at different educational stages, we observe that special education teachers have the lowest levels, possibly because they are the ones who encounter more difficult situations of different kinds, which would point to the need to receive greater professional support to strengthen their resilience.

Finally, the fact that teachers with more years of experience have a higher mean in resilience, although not a significant difference, may be due to the fact that they have had more opportunities in the school to develop resilience than other teachers with less experience.

## 5. Conclusions

The school is a very important community space where the links between community members are strengthened and the integral development of people is worked on, since they are not only spaces for the transmission of content, but also of values, habits and beliefs. Therefore, it is essential to focus on schools as contexts for the promotion of resilience. Moreover, as part of society, schools are not exempt from experiencing adverse situations [39].

Therefore, taking into account the importance of teachers being resilient professionals to promote the development of resilience in students and their own personal and occupational well-being, having valid instruments for measuring teacher resilience is an aspect of great importance for the educational field, since it will make it possible to evaluate the degree of resilience of teachers, which will help to assess whether it is necessary to carry out training programs.

In this sense, the CD-RISC-10 can contribute to assessing the effectiveness of training programs, since teacher resilience is related to specific training in this area [31,75].

In this regard, it is necessary for teachers to possess competencies as resilient professionals; for this, it is important that they have training in aspects related to the concept and models of resilience; characteristics and implications of a resilient teacher; protection and risk factors, both individual and contextual, that can influence the development of resilience; as well as knowing guidelines and strategies to intervene in the face of an adverse event [36].

In the present study, although the sample of participants is heterogeneous and includes teachers of pre-school, primary and special education, there is a limitation in that the selection of schools and teachers was not random, so that generalization of the results should be made with caution. Future studies on the role of resilience in teachers of Secondary Education and post-compulsory studies are suggested. Another limitation of our study is that it does not measure student resilience, so studies that measure resilience in teachers and their students are suggested to explore how the level of resilience of teachers influences the development and promotion of resilience in their students.

As a general conclusion, the importance of the research is highlighted, since it is the first empirical study on resilience carried out with a sample of teachers of Pre-school, Primary and Special Education. In addition, evidence is provided that the CD-RISC 10© scale has adequate psychometric properties and, therefore, can be used reliably and validly in the assessment of resilience in teachers.

## Figures and Tables

**Table 1:** Characteristics of participants.

		*n* = 290	%
Sex	Men	64	22.06%
	Women	226	77.93%
Type of school	Private school	15	5.17%
	Concerted school	52	17.93%
	Public school	223	76.89%
Educational stage	Early childhood education (3–6 years)	72	24.82%
	Primary education (6–12 years)	191	65.86%
	Special education	20	6.89%
Teaching experience	1–5 years	73	25.17%
	5–15 years	97	33.44%
	More than 15 years	120	41.37%

**Table 2:** Model fit indices.

Models	*χ* ^2^	*χ* ^2^_S-B_	*df*	CFI	NNFI	RMSEA
M1	549.21	45.67	33	0.97	0.98	0.036 [0.001, 0.060]
M2	206.469	39.19	35	0.70	0.66	0.130 [0.113, 0.148]

Note. *n* = 290; M1 (unifactorial model); M2 (bifactorial model); *χ*^2^ = chi square; *χ*^2^_S-B_ = Satorra–Bentler; *df* = degrees of freedom; CFI = comparative fit index; NNFI = non normed fit index; RMSEA = root mean square error of approximation with 90% confidence interval.

**Table 3:** Descriptive statistics and corrected item–total correlation for each item.

Items	M	DT	Asymmetry	Kurtosis	Corrected Correlation	α if Deleted
1. I am able to adapt to change	3.28	0.57	−0.12	−0.53	0.46	0.866
2. I can deal with whatever comes	3.10	0.66	−0.26	−0.25	0.56	0.862
3. I try to see the humorous side of problems	2.77	0.74	0.08	−0.60	0.37	0.868
4. Coping with stress can strengthen me	3.09	0.67	−0.17	−0.52	0.51	0.867
5. I tend to bounce back after illness or hardship	2.89	0.58	−0.08	0.11	0.44	0.862
6. I can achieve goals despite obstacles	2.98	0.71	−0.31	−0.10	0.40	0.863
7. I can stay focused under pressure	2.48	0.70	0.02	−0.22	0.48	0.863
8. I am not easily discouraged by failure	2.53	0.77	−0.02	0.10	0.44	0.866
9. I think of myself as astrong person	2.76	0.71	−0.15	0.18	0.61	0.861
10. I can handle unpleasant feelings	2.83	0.72	−0.22	0.16	0.49	0.863

**Table 4:** Correlations between the different items.

Items	1	2	3	4	5	6	7	8	9	10
1										
2	0.47 **									
3	0.29 **	0.21 **								
4	0.33 **	0.40 **	0.22 **							
5	0.22 **	0.24 **	0.27 **	0.30 **						
6	0.16 **	0.26 **	0.20 **	0.22 **	0.28 **					
7	0.23 **	0.38 **	0.19 **	0.24 **	0.35 **	0.29 **				
8	0.24 **	0.26 **	0.27 **	0.26 **	0.21 **	0.21 **	0.26 **			
9	0.32 **	0.15 **	0.23 **	0.45 **	0.27 **	0.32 **	0.40 **	0.42 **		
10	0.28 **	0.40 **	0.20 **	0.38 **	0.28 **	0.22 **	0.27 **	0.27 **	0.38 **	

Note: ** *p* < 0.01, two tails.

**Table 5:** Correlations (Pearson r) between CD-RISC-10 and C-DPR.

	CD-RISC-10	Factor 1: Beliefs	Factor 2: Knowledge	Factor 3: Resources	Total C-DPR
CD-RISC-10					
Factor 1: beliefs	0.564 **				
Factor 2: knowledge	0.790 **	0.389 **			
Factor 3: resources	0.809 **	0.415 **	0.556 **		
Total C-DPR	0.866 **	0.460 **	0.602 **	0.825 **	

Note: ** *p* < 0.01.

**Table 6:** Resilience level of teachers according to demographic variables.

		*n* = 290	M	SD
Sex	Men	64	29.03	3.89
	Women	226	28.47	4.08
Type of school	Private school	15	29.21	3.68
	Concerted school	52	28.96	4.07
	Public school	223	28.63	4.39
Educational stage	Early childhood education (3–6 years)	72	28.39	3.87
	Primary education (6–12 years)	191	29.24	3.56
	Special education	20	27.50	4.70
Teaching experience	1–5 years	73	28.57	4.45
	5–15 years	97	28.63	3.48
	More than 15 years	120	29.07	3.95

Note: M: Mean; SD: Standard Deviation.

## Data Availability

The datasets generated for this study are available from the corresponding author upon reasonable request.
